# Influence of ethanolic extract of avocado (*Persea americana* Mill.) seed flour on the estrous cycle, the serum concentrations of reproductive hormones, and the activities of oxidative stress markers in female cavies (*Cavia porcellus* L.)

**DOI:** 10.5455/javar.2021.h540

**Published:** 2021-09-20

**Authors:** Dongmo Nguedia Arius Baulland, Vemo Bertin Narcisse, Tchoffo Hervé, Mohamadou Adamou, Chongsi Margaret Mary Momo, Djuissi Motchewo Nadège, Mahamat Tahir Markhous Adam, Ngoula Ferdinand

**Affiliations:** 1 Department of Animal Sciences, Faculty of Agronomy and Agricultural Sciences, University of Dschang, Dschang, Cameroon; 2 Department of Biology, Faculty of Science and Techniques, University of Adam Barka of Abéché, Abéché, Chad

**Keywords:** Estrous cycle, female cavy, oxidative stress, *Persea americana* seed, reproduction

## Abstract

**Objective:**

The purpose of this study was to determine the effects of the ethanolic extract of avocado seed flour on the estrous cycle characteristics, the concentrations of reproductive hormones [luteinizing hormone (LH) and estradiol], and the activities of some tissues (ovarian and uterine) that are markers of oxidative stress in female guinea pigs (*Cavia porcellus*).

**Materials and Methods:**

Twenty-four female cavies with normal estrous cycles and equivalent body weights (464.25 and 71.88 gm) were randomly assigned to four groups, each with six females. The control group received 1 ml of distilled water orally, whereas the EE100, EE200, and EE400 groups received 100, 200, and 400 mg/kg bw of ethanolic extract of *Persea americana* seed, respectively. Following that, three consecutive estrous cycles were observed using vaginal smears. After the trial, the females were slaughtered, and blood and organs were gathered for study.

**Results:**

The mean duration of the estrus phase is considerably (*p* < 0.05) longer in group EE100 animals than in control animals. LH concentrations were substantially (*p* < 0.05) higher in females in group EE200 than in controls. Total cholesterol levels typically dropped in females in the extract groups, but only significantly (*p* < 0.05) in those in group EE100 compared to the control group. Serum estradiol levels increased considerably (*p* < 0.05) in treated females compared to controls. Catalase activity rose considerably (*p* < 0.05) in the ovaries of group EE400 when compared to the control. Superoxide dismutase activity rose considerably (*p* < 0.05) in the uterus of female cavies given the extract compared to the control.

**Conclusion:**

Avocado seed ethanolic extract prolongs the estrus phase, increases estradiol and LH levels, and protects the uterus from oxidative stress in female cavies.

## Introduction

Reproduction is a biological process that results in the formation of new individuals within a species from previously existing individuals [1,2]. Numerous factors can lead to an animal’s inability to reproduce, including endocrine/metabolic illnesses and oxidative stress, which is particularly frequent in females during heat and gestation. These latter conditions are the major causes of infertility and low fertility [3,4]. Natural substances such as whole plants, plant parts, extracts from plants, and essential oils from plants have been shown to be an effective and long-lasting solution to this condition. Indeed, they include bioactive chemicals that endow them with a range of pharmacological properties recognized by classical pharmacopeia [5]. One of these plants is avocado.

*Persea americana* Mill. is a North American natural plant. The Lauraceae family includes this tasty fruit tree. Indeed, the fruit of the avocado (avocado) contains a seed. Melgar et al. [6], Talabi et al. [7], Dabas et al. [8], and numerous other authors have demonstrated that avocado seeds may be a source of bioactive secondary metabolites such as phytosterols, polyphenols (flavonoids, triterpenoids, alkaloids, saponins, and tannins), and vitamins (A, C, and E), all of which have a variety of pharmacological properties. In this regard, Vinha et al. [9] and Mainul and Atif [10] showed that administering the ethanolic extract of avocado seed to rats generates an antioxidant effect. Abdel-Moneim et al. [11] reported that hydroethanolic extracts of the avocado fruit and seed exerted antioxidant and anti-inflammatory properties in Wistar rats exposed to diethylnitrosamine/2-acethylaminofluorene-induced cardio-nephrotoxicity. Imafidon and Okunrobo [12] demonstrated that the aqueous extract of avocado seed had hypotensive, hypoglycemic, and hypocholesterolemic effects in separate groups of hypertensive, hyperglycemic, and hypercholesterolemic albino rats. Similarly, Daramola et al. [13] found that phenolic compounds in *P. americana* seeds can protect spermatozoids present in goat sperm from the deleterious effects of free radicals during sperm cryopreservation. Mvondo et al. [14] reported that the ethanol extract of avocado seed powder inhibited the proliferation of uterine endometrial hyperplasia produced by tamoxifen in Wistar rats. Minko et al. [15] showed in a rat model of endometriosis that the ethanolic extract of *P. americana* seeds successfully induces endometrial implant regression and recovers the ovarian dynamic.

However, with the exception of the study conducted by Tatsinkou et al. [16], which showed that the aqueous extract of avocado seeds administrated to guinea pigs induced an increase in the weight of young at birth and weaning, to our knowledge, no other study has focused on the use of avocado seed extracts in healthy animals with a view of improving their reproduction. That is why this study was initiated with the aim of contributing to the amelioration of the reproduction of female mammals using avocado seed. Specifically, it was to determine in female guinea pigs (*Cavia porcellus*) the effects of the ethanolic extract of avocado seed flour on the estrous cycle, the concentrations of reproductive hormones [Luteinizing hormone (LH), estradiol], and the activities of some tissues (ovarian and uterine) markers of oxidative stress.

## Materials and Methods

### Ethical approval

The experimental protocols for this study were approved by the Ethical Committee of the Department of Animal Science of the Faculty of Agronomy and Agricultural Sciences of the University of Dschang, Cameroon, and were conducted in strict accordance with internationally accepted standard ethical guidelines for laboratory animal use and care as described in the European Union guidelines; EEC Directive 86/609/EEC, of the 24th November 1986.

### Animal material

Twenty-four adult female guinea pigs (English breed) were utilized in this study. They were obtained from the Teaching and Research Farm of the University of Dschang’s Faculty of Agronomy and Agricultural Sciences. They had similar body weights (464.25 and 71.88 gm).

### Plant material

Mature fruits of *P. americana* (avocados) were picked from the same tree in Bangang, a location in Mbouda in the Bamboutos division of Cameroon’s West region. Following that, the plant was validated at the Yaoundé National Herbarium (Cameroon) under the number 18604/SRF/CAM, using the Daniel Dong number 80 as a comparative model. Following that, these fruits were cleaned and the extracted seeds were crushed into fine particles, before being directly boiled in a constant pressure pot (100°C for 15 min) and dried under shade, as described by Talabi et al. [7]. This treatment aimed to reduce the cyanidric acid concentration in the avocado seed. The dried smashed seed were milled to obtain a powder. Finally, the resulting homogenous powder was used to prepare the ethanolic extract.

### Preparation of the ethanolic extract of avocado seeds

The ethanolic extract of avocado seeds was prepared using a slightly modified version of the maceration process described by Egbuonu et al. [17]. Exactly 500 gm of avocado seed flour was added to 2,000 ml of 100% ethanol and regularly mixed for 3 days. Following this time period, the resulting homogenate was filtered using Whatman No. 1 filter paper. Following that, the filtrate was concentrated in a rotary evaporator (at 60°C) and then stored in opaque glass bottles at 4°C until use.

### Lodging and feeding

This experiment utilized 24 adult female guinea pigs with normal estrous cycles. They were sorted into four groups of six females each and housed in four cages with six cavies each until the test concluded. Each of these cages had a feeder and a waterer. Prior to introducing the females, all cages were disinfected. Throughout the experimental period, each group received *ad libitum* potable water and compounded feed made with ingredients obtained from a local market, except for the *Pennisetum purpureum*, which was harvested on the Teaching and Research Farm of the Faculty of Agronomy and Agricultural Sciences of the University of Dschang. This basic ration had 97.82% dry matter, 86.06% organic matter, 16.79% crude protein, 15.80% crude fiber, 13.94% ash, and 2,750 kcal/kg metabolizable energy.

### Experimental protocol

After 2 weeks of adaption, 4 successive estrous cycles were seen in 40 female guinea pigs for roughly 68 days (17 days per cycle). Exactly 24 female cavies with normal cyclicity were utilized to test the effects of an ethanol extract of *P. americana* seed flour on several reproductive parameters. These cavies were weighed on a scale with a capacity of 5 kg and a sensitivity of 1 gm, and then randomly assigned to four groups of six cavies each that were comparable in body weight (bw), and put in a completely randomized configuration. Each of the four groups received a unique treatment. Each female was identified by an ear tag bearing its unique code and was treated as an experimental unit. These females were gavaged throughout the test with 1 ml of distilled water for the control group and 100, 200, or 400 mg/kg bw of ethanolic extract of *P. americana* seed for the EE100, EE200, and EE400 groups, respectively. The varied solutions for administration were made by adding 2.5, 5, and 10 mg of the extract individually into three 50 ml volumetric flasks and filling to volume with distilled water to obtain concentrations of 50, 100, and 200 mg/ml for dosages of 100, 200, and 400 mg/ml, respectively. These solutions were kept in dark glass vials and administered to the subjects daily (between 6 and 8 a.m.). Following that, vaginal smears were taken for roughly 51 days, corresponding to three estrous cycles, on the same cavies (between 8 and 9 a.m.). Throughout the test period, the amounts of solutions to be administered were changed weekly based on the weight of the females.

### Realization of vaginal smears

Each guinea pig’s vaginal contents were collected daily (between 8 and 9 a.m.) using a glass pipette containing 250 l of NaCl 0.9%, spread on a slide, and left at room temperature for a few minutes to allow the sample to stick effectively to the microscope slide. For 5 min, the sample was fixed in 70% methanol and then stained with methylene blue (0.5%) for 10 min. The slide was then cleaned with clean water to remove any remaining dye [18]. The different phases of the estrous cycle were determined by analyzing the proportion of the three distinct cell types present on the slide under an optical microscope (at magnifications of 100 and 400). The proestrus phase is defined by the predominance of round nucleated epithelial cells; the estrus phase is defined by the abundance of non-nucleated cornified epithelial cells; the metestrus phase is defined by the presence of round nucleated, non-nucleated cornified epithelial cells, and small round leucocytes in equal proportions; and the diestrus phase is defined by the predominance of leucocytes [19].

### Samples’ collection and biochemical analysis

Six female cavies from each group were sedated with ethyl ether and slaughtered during the estrous phase immediately following the third estrous cycle. Blood was obtained from the jugular vein in dry tubes during this exercise (free from anticoagulant). After centrifuging the blood sample at 3,000 rpm for 5 min, the serum was extracted and kept at −20°C until used. Total cholesterol was determined using the Chronolab kit, while estradiol and LH were determined using enzyme-linked immunosorbent assay kits from DRG Diagnostics and Omega Diagnostics, respectively. Following laparotomy, many organs (ovaries and uterus) were removed, weighed, and the uterus’ length, width, and thickness were measured using a vernier caliper. Each cavy’s left ovary and uterine horn were crushed separately in porcelain mortars with Tris buffer (pH 7.4) until a 15% homogenate was obtained. After 30 min of spinning at 3,000 rpm (using a cold centrifuge), the supernatant was collected into tiny tubes and stored at −20°C in the freezer. This homogenate was used to determine the doses of oxidative stress markers malondialdehyde (MDA), superoxide dismutase (SOD), catalase (CAT), and total peroxidases (POX) using the methods described by Nilsson et al. [20], Misra and Fridovich [21], Sinha [22], and Habbu et al. [23], respectively, as well as the dosages of ovarian and uterine total proteins using the Chronolab kit.

### Statistical analysis

Statistical analyses were conducted using Statistical Package for the Social Sciences 25.0 software. The obtained data were subjected to one-way analysis of variance to test the effects of different doses of ethanolic extract of avocado seed on the studied parameters. Duncan’s *post-hoc* test was used to separate means when the differences were significant. The limit of signification was 5% and the results obtained were expressed as mean ± standard deviation.

## Results

### Phytochemical composition

The results of the phytochemical analysis of the ethanolic extract of avocado seed powder are presented in Table 1.

### Influence of the ethanol extract of avocado seed flour on the mean duration of the different phases of the estrous cycle of guinea pigs

**Figure 1.**
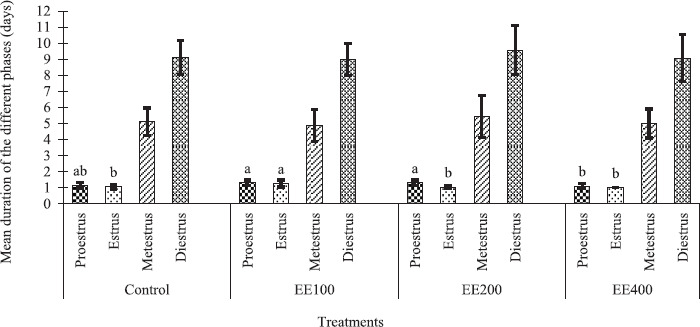
Influence of the ethanolic extract of *P. americana* seed flour on the mean duration of the phases of the estrous cycle in guinea pigs. (^a, b^) means with different letters are significantly different (*p <* 0.05). Control = 1 ml/kg bw of distilled water; EE100, EE200, and EE400 = ethanolic extract of *P. americana* seed flour at the doses of 100, 200, and 400 mg/kg bw, respectively.

As shown in Figure 1, different doses of ethanolic extract of *P. americana* seed had no significant influence (*p >* 0.05) on the duration of the proestrus phase in guinea pigs when compared to the control group. However, when only animals treated to an ethanolic extract of avocado seed were examined, the duration of the proestrus phase was significantly (*p <* 0.05) shorter in females from group EE400 than from groups EE100 and EE200. The extract significantly increases the mean duration of estrus phase (*p <* 0.05) in cavies in group EE100 when compared to control and other treatment groups. On the contrary, the avocado seed extract had no significant effect on the mean lengths of the metestrus and diestrus phases (*p >* 0.05).

**Table 1.** Phytochemical composition of the ethanolic extract of avocado seed powder.

Bioactive compound	Present/absent
Flavonoids	+
Alkaloids	−
Phenols	++
Triterpens	+
Steroids	+
Saponins	++
Tannins	++

−: absent; +: present; ++: present in height quantity.

### Influence of the ethanol extract of P. americana seed flour on the estrous cycle duration in cavies

**Figure 2.**
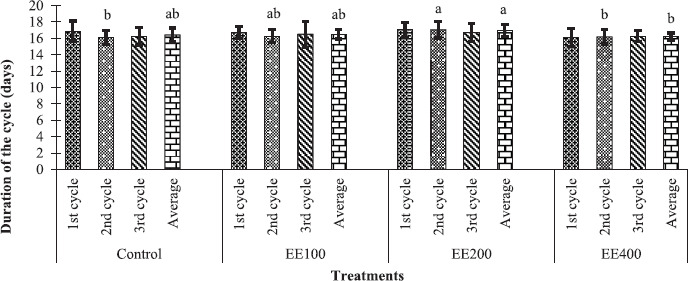
Variations in the length of the estrous cycle as a function of the different doses of ethanolic extract of *P. americana* seed flour in guinea pigs. (^a, b^) means with different letters are significantly different (*p <* 0.05). Control = 1 ml/kg bw of distilled water; EE100, EE200, and EE400 = ethanolic extract of *Persea americana* seed flour at the doses of 100, 200, and 400 mg/kg bw, respectively.

Figure 2 shows that the various doses of ethanolic extract of *P. americana* seed had no significant effect (*p >* 0.05) on the first, third, and average of the three cycles in cavies when compared to the control group’s females. In comparison to the control and EE400 groups, the ethanolic extract of avocado seeds at 200 mg/kg bw significantly extended (*p <* 0.05) the duration of the second cycle in cavies. When only the treatment groups were evaluated, the average duration of the three cycles was substantially (*p <* 0.05) longer in females in group EE200 than in females in group EE400.

### Influence of the ethanol extract of P. americana seed flour on the relative weights of the ovaries, uterus, and uterine mensurations in guinea pigs

As shown in Table 2, females treated with avocado seed extract had a drop in ovary weight, although the decrease was significant (*p <* 0.05) only in females in the EE400 group compared to the control group. In comparison to the control group, the avocado seed extract had no significant (*p >* 0.05) effect on the relative weight, length, width, and thickness of the uterus in females.

**Table 2.** Influence of the ethanolic extract of *P. americana* seed flour on the relative weights of the ovaries, uterus, and uterine mensurations in guinea pigs.

Weights of ovaries, uterus (mg/100 gm bw), and uterus mensurations (cm)	Control (*n* = 6)	Doses of ethanolic extract of *P. americana* seed powder (mg/kg bw)			*p-*value
		EE100 (*n* = 6)	EE200 (*n* = 6)	EE400 (*n* = 6)	
Weights of organs					
Ovaries	10.89 ± 2.53^a^	10.52 ± 2.71^ab^	10.07 ± 1.20^ab^	07.91 ± 1.97^b^	0.04
Uterus	213.13 ± 33.40	201.78 ± 37.84	225.02 ± 36.52	238.78 ± 54.92	0.47
Uterus mensurations					
Length	4.72 ± 0.73	4.63 ± 0.62	5.02 ± 0.78	4.72 ± 0.76	0.81
Width	0.75 ± 0.05	0.72 ± 0.07	0.80 ± 0.09	0.77 ± 0.08	0.32
Thickness	0.63 ± 0.07	0.55 ± 0.08	0.60 ± 0.09	0.59 ± 0.07	0.39

(^a,b^) means with different letters are significantly different (*p <* 0.05). *n* = number of samples; *p* = probability; Control = 1 ml/kg bw of distilled water; EE100, EE200, and EE400 = ethanol extract of *P. americana* seed flour at the doses of 100, 200, and 400 mg/kg bw, respectively.

### Influence of the ethanol extract of P. americana seed flour on the serum concentration of total cholesterol, estradiol, and LH in female guinea pigs

Avocado seed extract at various doses generally resulted in a decrease in serum total cholesterol content. However, the decrease was only significant (*p <* 0.05) in cavies belonging to group EE100 when compared to those belonging to the control group (Table 3). On the contrary, the various dosages of extract boosted LH levels in female cavies. When females receiving 200 mg/kg bw were compared to those in the control group, the increase was significant (*p <* 0.05). Serum estradiol concentrations increased considerably (*p <* 0.05) in all females treated with ethanolic extract of *P. americana* seeds when compared to the control group.

### Influence of the ethanol extract of P. americana seed flour on ovarian and uterine markers of oxidative stress in cavies

**Case of the ovaries**

The ethanolic extract of *P. americana* seeds at 200 mg/kg bw significantly increased the content of total proteins in the ovaries of cavies (*p <* 0.05) when compared to the control group. Animals treated with avocado seed extract had an increase in CAT activity. Meanwhile, only females receiving 400 mg/kg pc of extract had a significant increase (*p <* 0.05) in comparison to the control group. The therapy had no significant effect on the ovarian activity of MDA, SOD, or POX (*p >* 0.05) (Table 4).

**Table 3.** Influence of the ethanolic extract of *P. americana* seed flour on serum concentrations of total cholesterol, LH, and estradiol in female guinea pigs.

Biochemical parameters	Control (*n* = 6)	Doses of ethanolic extract of *P. americana* seed powder (mg/kg bw)			*p-*value
		EE100 (*n* = 6)	EE200 (*n* = 6)	EE400 (*n* = 6)	
Total cholesterol (mg/dl)	62.19 ± 6.53^a^	46.14 ± 8.47^b^	61.44 ± 11.42^a^	60.75 ± 7.12^a^	0.02
LH (mUI/ml)	5.63 ± 1.29^b^	7.75 ± 0.96^b^	14.50 ± 3.94^a^	7.80 ± 1.30^b^	0.00
Estradiol (pg/ml)	80.00 ± 14.14^c^	117.80 ± 14.46^b^	145.00 ± 7.07^a^	108.00 ± 11.51^b^	0.00

(^a, b, c^) means with different letters are significantly different (*p <* 0.05). *n* = number of samples; *p* = probability; Control = 1 ml/kg bw of distilled water; EE100, EE200, and EE400 = ethanol extract of *Persea americana* seed flour at the doses of 100, 200, and 400 mg/kg bw, respectively.

**Table 4.** Influence of the ethanolic extract of *Persea americana* seed flour on oxidative stress markers in the ovaries of guinea pigs.

Oxidative stress markers	Control (*n* = 6)	Doses of ethanolic extract of *P. americana* seed flour (mg/kg bw)			*p-*value
		EE100 (*n* = 6)	EE200 (*n* = 6)	EE400 (*n* = 6)	
Total proteins (gm/dl)	1.36 ± 0.09^b^	1.60 ± 0.21^ab^	1.88 ± 0.30^a^	1.67 ± 0.28^ab^	0.03
MDA (μM)	0.53 ± 0.06	0.61 ± 0.29	0,63 ± 0.12	0.73 ± 0.25	0.44
SOD (U/min/gm of ovarian proteins)	0.83 ± 0.23	0.89 ± 0.09	0.81 ± 0.09	0.84 ± 0.10	0.84
CAT (μM/min/gm of ovarian proteins)	1.52 ± 0.93^b^	2.04 ± 0.60^ab^	2.04 ± 0.60^ab^	3.18 ± 1.09^a^	0.04
POX (mM/min/gm of ovarian proteins)	6.17 ± 2.80	7.60 ± 3.96	6.83 ± 3.98	8.18 ± 2.32	0.80

(^a, b^) means with different letters are significantly different (*p <* 0.05). *n* = number of samples; *p* = probability; Control = 1 ml/kg bw of distilled water; EE100, EE200, and EE400 = ethanol extract of *P. americana* seed flour at 100, 200, and 400 mg/kg bw, respectively. MDA = malondialdehyde; SOD = superoxide dismutase; CAT = catalase; POX = total peroxidase.

**Table 5.** Influence of the ethanolic extract of *P. americana* seed flour on oxidative stress markers in the uterus of guinea pigs.

Oxidative stress markers	Control (*n* = 6)	Doses of ethanolic extract of *P. americana* seed flour (mg/kg bw)			*p-*value
		EE100 (*n* = 6)	EE200 (*n* = 6)	EE400 (*n* = 6)	
Total proteins (gm/dl)	1.74 ± 0.18	1.58 ± 0.10	1.79 ± 0.38	1.57 ± 0.25	0.36
MDA (μM)	1.27 ± 0.59	1.06 ± 0.30	0.91 ± 0.17	1.02 ± 0.12	0.36
SOD U/min/gm of uterine proteins)	0.77 ± 0.09^b^	0.91 ± 0.03^a^	0.93 ± 0.07^a^	0.91 ± 0.05^a^	0.00
CAT (μM/min/gm of uterine proteins)	1.23 ± 0.21	1.34 ± 0.11	1.17 ± 0.18	1.35 ± 0.13	0.20
POX (mM/min/gm of uterine proteins)	24.89 ± 9.69	22.57 ± 5.78	22.65 ± 8.08	21.86 ± 2.99	0.89

(^a, b^) means with different letters are significantly different (*p <* 0.05). *n* = number of samples; *p* = probability; Control = 1 ml/kg bw of distilled water; EE100, EE200, and EE400 = ethanol extract of *P. americana* seed flour at 100, 200, and 400 mg/kg bw, respectively. MDA = malondialdehyde; SOD = superoxide dismutase; CAT = catalase; POX = total peroxidase.

**Case of the uterus**

Except for SOD activity, which was significantly (*p <* 0.05) increased in the uterus of females treated with ethanolic extract of *P. americana* seeds compared to the control group, the uterine concentrations of total proteins, MDA, CAT, and POX activities were not significantly (*p >* 0.05) affected by the treatment (Table 5).

### Influence of the ethanol extract of P. americana seed flour on serum protein concentrations in female guinea pigs

According to Table 6, the various doses of *P. americana* seed extract had no significant influence (*p >* 0.05) on the serum concentrations of total proteins, albumin, and globulin in female cavies compared to the control group.

## Discussion

The present study discovered that the ethanolic extract of avocado (*P. americana* Mill.) seeds powder contains bioactive compounds such as flavonoids, phenols, triterpenes, steroids, saponins, and tannins. These findings corroborate those of Tremocoldi et al. [24], Bahru et al. [25], and a large number of other authors. According to numerous experts, these compounds contain a variety of pharmacological effects (antioxidants, antibacterial, anti-inflammatory, and so on) and can increase animal reproductive performances [10–13].

**Table 6.** Influence of the ethanolic extract of *P. americana* seed flour on the serum concentration of total proteins in female guinea pigs.

Proteins concentrations (gm/dl)	Control (*n* = 6)	Doses of ethanolic extract of *Persea americana* seed flour (mg/kg bw)			*p-*value
		EE100 (*n* = 6)	EE200 (*n* = 6)	EE400 (*n* = 6)	
Total proteins	4.40 ± 0.33	4.11 ± 0.36	4.20 ± 0.55	4.23 ± 0.65	0.79
Albumin	2.49 ± 0.50	2.51 ± 0.28	2.51 ± 0.27	2.63 ± 0.25	0.90
Globulin	1.91 ± 0.34	1.60 ± 0.19	1.68 ± 0.37	1.60 ± 0.39	0.36

(^a, b^) In the same row, means with different letters are significantly different (*p <* 0.05). *n* = number of samples; *p* = probability; Control = 1 ml/kg bw of distilled water; EE100, EE200, and EE400 = ethanol extract of *P. americana* seed flour at the doses of 100, 200, and 400 mg/kg bw, respectively.

On the basis of these features, the current study examines the effects of an ethanolic extract of *P. americana* seed flour on female mammalian reproduction.

The estrous cycle is a series of anatomical, physiological, and behavioral changes that occur in a female mammal between two estrus. It is classified into four phases: proestrus, estrus, metestrus, and diestrus. The estrus phase, or heat phase, is when the female accepts mating. Estrous cycles are regulated by reproductive hormones such as LH, follicle-stimulating hormone (FSH), and estrogens [26,27]. According to the results of this study, the ethanolic extract of *P. americana* seed powder prolongs the proestrus and estrus phases in cavies. Additionally, it elevates the serum levels of LH and estrogen in females treated. These findings corroborate those of Koneri et al. [28], who administered 250 and 500 mg/kg bw of ethanolic extract of *Momordica cymbalaria* roots to Wistar rats over a 15-day period; Wijayanti et al. [29] administered 10, 50, and 90 mg/kg bw of ethanolic extract of *Anredera cordifolia* leaf to female guinea pigs from 10 days prepartum. Ganguly et al. [30] administrated 300 mg/kg bw of methanolic extract of Mimosa pudica root for 21 consecutive days to albino mice with regular estrous cycle. However, it contrasts the results reported by Nayanatara et al. [31] after administering an aqueous extract of *Cynodon dactylon* to female Wistar rats via gavage for 30 days at a concentration of 400 mg/kg bw. Indeed, phytoestrogens (flavonoids, triterpenes, and sterols) found in certain plants, such as avocado seed, have similar molecular structures to estrogens, enables them to bind to estrogen receptors found in certain tissues and act as estrogen agonists or antagonists, depending on their concentrations and the tissues to which they bind. In the case of this study, they would have produced an increase in estrogen production (including estradiol) in response to an increase in blood levels of reproductive hormones (FSH and LH), thereby lengthening the proestrus and estrus phases of the estrous cycle. Indeed, during proestrus (the phase characterized by folliculogenesis), excessive FSH enhances the recruitment and development of primordial follicles until the preovulatory stage. Following that, the elevated levels of LH and estrogens triggered the rupture of a large number of mature follicles (increased ovulation rate) and prolonged the proestrus and estrus stages (heat) of the estrous cycle [32,33].

The process through which cholesterol is transformed to steroid hormones is called steroidogenesis, due to the fact that cholesterol is a precursor to steroid hormones such as estrogen and progesterone [2]. The observed decrease in total cholesterol levels in female cavies treated with *P. americana* seed extract demonstrates the plant’s hypocholesterolemic action in animals. This finding is consistent with that of Pahua-Ramos et al. [34], who administered 125, 250, and 500 mg/kg bw of methanol extract of avocado seeds powder to adult male mice over a 6-day period via gavage. Additionally, it corroborates the findings of Nwaoguikpe and Braide [35], who administered an aqueous extract of avocado seeds to rabbits orally for 2 months at doses of 100 and 200 mg/kg bw. This decrease in serum total cholesterol levels in female cavies may be because phytoestrogens in avocado seeds encouraged the use of cholesterol in the blood as a raw material for estrogen and progesterone production during ovarian steroidogenesis [2]. They subsequently had an effect on the serum estradiol level. According to Saravanan and Ignacimuthu [36], this result could also be explained by the fact that saponins, which are abundant in plants such as avocado seeds and have the ability to lower blood cholesterol levels by interacting with bile acids to form large mixed micelles, may be responsible for the decrease in total cholesterol concentrations observed in female cavies treated with avocado seed extract.

Female mammals are subjected to physiologically induced oxidative stress during reproduction (estrus, gestation, birth, and lactation) [1]. Oxidative stress is a condition in which the antioxidant and reactive oxygen species (ROS) systems are out of balance, favoring the latter. When cells are stressed, ROS cause denaturation of the DNA and lipid peroxidation of the cell membranes. They have been shown to negatively affect physiological processes ranging from oocyte maturation to fertilization, pregnancy, and embryo development [37]. Under stressful conditions, MDA (a byproduct of lipid peroxidation) and enzymatic antioxidants such as SOD, POX, and CAT levels increase. Antioxidants provide protection against free radicals [37]. According to the findings of this investigation, the ethanolic extract of avocado seed increased the concentrations of total proteins, CAT, MDA, and POX activities in ovarian tissue. Indeed, this increase in MDA activity in female cavies treated with extracts may account for the ovary’s relative weight loss due to lipid peroxidation caused by oxidative stress [38]. Different doses of avocado seed extract resulted in a non-significant drop in MDA activity and a substantial increase in SOD activity in uterine tissue. However, this treatment had no discernible influence on the uterus’ relative weight, length, width, or thickness.

This finding corroborates that of Mahadeva et al. [39], who administered 300 mg/kg bw of aqueous extract of *P. americana* seed to hyperglycemic Wistar rats for 30 days. The ethanolic extract of *P. americana* seed flour may not have provided adequate protection against free radicals in the ovarian tissue. Indeed, during the heat phase, ovulation, and steroidogenesis that occur in the ovaries, the P450 system and inflammation produce more ROS, rendering antioxidants incapable of neutralizing them [40]. On the other hand, avocado seed extract shielded the uterus from the adversity caused by ROS. Antioxidant compounds found in the avocado seed, such as flavonoids, polyphenols, vitamin C, and E, may have contributed to this outcome [41].

Proteins are a class of molecules that serve a variety of tasks in an organism. They have been shown to function as hormone transporters, cellular receptors, enzymes, and antibodies [42]. Avocado seed extract had no significant effect on the serum protein levels in cavies. Indeed, phenolic substances such as flavonoids found in avocado seeds may have reduced protein denaturation caused by ROS (loss of structure and function), so balancing the rates of anabolism and catabolism [43].

It would have been beneficial to determine the effects of the ethanolic extract of avocado *P. americana* seed flour on actual fertility in female cavies or to isolate the various bioactive molecules contained in this extract and evaluate their individual and combined effects on those parameters during this study.

## Conclusion

The following findings were reached at the conclusion of this study, which highlights the effects of ethanolic extract of *P. americana* seed flour on the estrous cycle features, reproductive hormone levels, and oxidative stress markers in the ovary and uterus of female cavies. The ethanolic extract of avocado seed powder resulted in an increase in the duration of heat (estrus phases) in female cavies receiving dosages of 100 and 200 mg/kg bw, a decrease in blood total cholesterol, and an increase in serum LH and estradiol concentrations in female cavies. Additionally, it protects the uterus from free radical damage.

## List of Abbreviations

CAT: catalase; DEN: diethylnitrosamine; DNA: deoxyribonucleic acid; FSH: follicle-stimulating hormone; LH: luteinizing hormone; MDA: malondialdehyde; POX: total peroxidase; ROS: reactive oxygen species; SOD: superoxide dismutase.

## References

[1] Zelena D (2015). The janus face of stress on reproduction: from heath to disease. Int J Endocrinol.

[2] Gayrard V (2007). Physiologie de la reproduction des mammifères. Polycopié. Unité de physiologie-physiopathologie.

[3] Gillet A, Deflers H, Marlier D (2016). Les kystes ovariens chez le cobaye. Rev Med Vet.

[4] Vannuccini S, Clifton VL, Fraser IS, Taylor HS, Critchley H, Giudice LC (2016). Infertility and reproductive disorders: impact of hormonal and inflammatory mechanisms on pregnancy outcome. Hum Reprod Updates.

[5] Ramawat KG, Dass S, Mathur M, Ramawat K (2009). The chemical diversity of bioactive molecules and therapeutic potential of medicinal plants. Herbal drugs: ethnomedicine to modern medicine.

[6] Melgar B, Dias MI, Ciric A, Sokovic M, Garcia-Castello EM, Rodriguez-Lopez AD (2018). Bioactive characterisation of *Persea americana* Mill. By-producs: a rich source of inherent antioxidants. Ind Crops Prod.

[7] Talabi J, Olukemi AO, Ajayi OO, Adegoke GO (2016). Nutritional and antinutritional compositions of processed avocado (*Persea americana* Mill) seeds. Asian J Plant Sci Res.

[8] Dabas D, Shegog RM, Ziegler GR, Lambert JD (2013). Avocado (*Persea americana*) seed as a source of bioactive phytochemicals. Curr Pharm Des.

[9] Vinha AF, Moreira J, Barreira SVP (2013). Physicochemical parameters, phytochemical composition and antioxidant activity of the algarvian avocado (*Persea americana* Mill.). J Agric Sci.

[10] Mainul H, Atif B (2011). Insuline stimulative and anti-oxidative effects of *Persea americana *fruit extract on streptozotocin induiced hyperglycemic rats. J Med Biol Sci.

[11] Abdel-Moneim AA, Osama MA, Hanaa IF, Eman EM (2017). The preventive effects of avocado fruit and seed extracts on cardio-nephrotoxicity induced by diethylnitrosamine/2-acetylaminoflurine in Wistar rats. Basic Sci Med.

[12] Imafidon EK, Okunrobo OL (2009). Biochemical evaluation of the tradomedicinal use of seeds of *Persea americana* Mill., (family: Lauraceae). World J Med Sci.

[13] Daramola JO, Onanuga OD, Abioja MO, Adeleke M, Olowofeso O, Oke OE (2016). Effects of avocado seed extract in different trisextenders on sperm and oxidative stress indices of vitrified goat spermatozoa. J Agric Sci.

[14] Mvondo MA, Messongue MN, Djamen D (2020). The ethanol extract of avocado [*Persea americana* Mill. (*Lauraceae*)] seeds reduced the hyperplastic effect of tamoxifen on uterine endometrium without changing its effect on the mammary gland. Adv Trad Med.

[15] Minko ES, Mvondo MA, Ngadjui E, Kemka NFX, Watcho P (2020). The ethanol extract of avocado (*Persea americana* Mill. (*Lauraceae*)) seeds successfully induces implant regression and restores ovarian dynamic in a rat model of endometriosis. Evid *Based* Complement Alternat Med.

[16] Tatsinkou AS, Miegoue E, Noumbissi MNB, Sawa C, Mube HK, Nguedia G (2020). Effet des extraits de noyaux d’avocat sur la croissance et la variation du microbiote caecal chez le cochon d’Inde (*Cavia porcellus*). Int J Biol Chem Sci.

[17] Egbuonu AC, Omodamiro OD, Achi NK, Opara CI (2018). Effect of ethanolic extract of avocado pear (*Persea americana*) seed on normal and monosodium glutamate-compromised rats’ kidney histology and serum bio-functional parameters. EC Pharmacol Toxicol.

[18] Sahar O, Abeer AAES (2007). Modified vaginal smear cytology for the determination of the rat estrous cycle phases, versus ordinary Papanicolaou technique, verifie by light and scanning electron microscopic examination of the endometrium. Egypt J Histol.

[19] Marcondes FK, Bianchi FJ, Tanno AP (2002). Determination of the estrous cycle phases of rats: some helpful considerations. Braz J Biol.

[20] Nilsson UA, Olsson LI, Carlin G, Bylund-Fellenius AC (1989). Inhibition of lipid peroxidation by spin labels. J Biol Chem.

[21] Misra HP, Fridovich I (1972). The generation of superoxide radical during the autoxidation of hemoglobin. J Biol Chem.

[22] Sinha AK (1972). Colorimetric assay of catalase. Anal Biochem.

[23] Habbu PV, Shastry RA, Mahadevan KM, Hanumanthachar J, Das SK (2008). Hepatoprotective and antioxidant effects of *Argyreia speciosa* in rats. Afr J Trad Compl Altern Med.

[24] Tremocoldi MA, Rosalen PL, Franchin M, Massarioli AP, Denny C, Daiuto ER (2018). Exploration of avocado by-products as natural sources of bioactive compounds. PLoS One.

[25] Bahru TB, Tadele ZH, Ajebe EG (2019). A review on avocado seed: functionality, composition, antioxidant and antimicrobial properties. Chem Sci Int J.

[26] Gnanagurudasan E, Senthil KSK, Leena DJ (2017). Comparative study on the estimation of estrous cycle in mice by visual and vaginal lavage method. J Clin Diag Res.

[27] Cora MC, Kooistra L, Travlos G (2015). Vaginal cytology of the laboratory rat and mouse: review and criteria for the staging of the estrous cycle using stained vaginal smears. Toxicol Pathol.

[28] Koneri R, Balaraman R, Saraswati CD (2006). Antiovulatory and abortifacient potential of ethanolic extract of roots of *Momordica cymbalaria* Fenzl. in rat. Indian J Pharmacol.

[29] Wijayanti D, Setiatin ET, Kurnianto E (2017). Study on postpartum estrus of guinea pigs (*Cavia cobaya*) using *Anredera cordifolia* leaf extract. Vet World.

[30] Ganguly M, Devi N, Mahanta R, Borthakur MK (2007). Effect of *Mimosa pudica* root extract on vaginal estrous and serum hormones for screening of antifertility activity in albino mice. Contraception.

[31] Nayanatara AK, Akshatha A, Sharannya K, Anwar AS, Rejecsh EP, Bhagyalak S (2012). Effect of *Cynodon*
*dactylon* extract on estrous cycle and reproductive organs in female wistar rats. Int J Anal Pharm Biomed Sci.

[32] Retana-Márquez S, Hernández H, Flores JA, Muñoz-Gutiérrez M, Duarte G, Vielma J (2012). Effects of phytoestrogens on mammalian reproductive physiology. Trop Subtrop Agroecosyst.

[33] Ososki AL, Kennelly EJ (2003). Phytoestrogens: a review of the present state of research. Phytother Res.

[34] Pahua-Ramos ME, Ortiz-Moreno A, Chamorro-Cevallos G, Hernández-Navarro MD, Garduño-Siciliano L, Necoechea-Mondragón H (2012). Hypolipidemic effect of avocado (*Persea americana* Mill.) seed in a hypercholesterolemic mouse model. Plant Foods Hum Nutr.

[35] Nwaoguikpe RN, Braide W (2011). The effect of acqueous extract of *Persea americana *(avocado pear) on serum lipid and cholesterol levels in rabbits. Afr J Pharm Pharmacol Res.

[36] Saravanan M, Ignacimuthu S (2015). Hypocholesterolemic effect of Indian medicinal plants-a review. Med Chem.

[37] Agarwal A, Gupta S, Sharma R (2005). Role of oxidative stress in female reproduction. Reprod Biol Endocrinol.

[38] Chongsi MMM, Ngoula F, Ngouateu KOB, Makona NAM, Kenfack A, Vemo BN (2019). Oxidative effects of potassium dichromate on biochemical, hematological characteristics, and hormonal levels in rabbit doe (*Oryctolagus cuniculus*). Vet Sci.

[39] Mahadeva RUS, Mainul H, Atif AB (2011). Insulin stimulative and anti-oxidative effects of *Persea americana* fruit extract on streptozotocin induced hyperglycemic rats. J Med Biol Sci.

[40] Fujii J, Iuchi Y, Okada F (2005). Fundamental roles of reactive oxygen species and protective mecanisms in the female reproductivesystem. Reprod Biol Endocrinol.

[41] Deutcheu NS, Ngoula F, Manfo TFP, Ngouateu KOB, Mabou NJL, Vemo B (2019). Oxidative stress and reproductive damage induced by lead acetate in female guinea pig (*Cavia porcellus*): curative effects of hydroethanolic extract of *Spirulina platensis*. Am J Anim Vet Sci.

[42] Tissier M (2011). Contribution à l’étude du stress oxydant chez le chien de cross canin. Thèse de Doctorat en Médecine Vétérinaire, Université Claude-Bernard-Lyon, Villeurbanne, France.

[43] Antasionasti I, Riyanto S, Rohman A (2017). Antioxidant activities and phenolics contents of avocado (*Persea americana *Mill.) peel *in vitro*. Res J Med Plants.

